# The Impact of Tobacco Smoking on Treatment Response Among Patients With Psoriasis Undergoing Biologic Treatment: Prospective Observational Study

**DOI:** 10.2196/90963

**Published:** 2026-03-04

**Authors:** Fanlingzi Shen, Yuning Ding, Xiuqi Zhang, Quanruo Xu, Zhen Duan, Ruiqi Cai, Rui Zhang, Xiangjin Gao, Ruiping Wang

**Affiliations:** 1 Clinical Research Center, Shanghai Skin Disease Hospital, 1278 Baode Road, Shanghai, Shanghai, 200443, China, 86 13816845803; 2 School of Medicine, Tongji University, Shanghai, Shanghai, China; 3 School of Public Health, Shanghai University of Traditional Chinese Medicine, Shanghai, Shanghai, China

**Keywords:** psoriasis, tobacco smoking, biologics, treatment response, Psoriasis Area and Severity Index, PASI

## Abstract

**Background:**

Tobacco smoking is viewed as a behavioral risk factor for psoriasis initiation and progress, even among those undergoing biologic treatment. However, evidence regarding the association between tobacco smoking and treatment response to biologics among patients with psoriasis is limited.

**Objective:**

This study aimed to explore the impact of tobacco smoking on the efficacy of biologic treatment in patients with psoriasis.

**Methods:**

Patients with psoriasis undergoing biologic treatment were recruited from 2022 to 2024 at the Shanghai Skin Disease Hospital. Demographic characteristics and smoking habits were collected using a structured questionnaire. Clinical features and treatment efficacy were assessed and recorded by dermatologists at baseline and weeks 4, 8, 12, 24, and 48 after treatment, and the Psoriasis Area and Severity Index (PASI) 75 and PASI 90 measures were calculated for treatment efficacy evaluation.

**Results:**

A total of 192 patients with psoriasis were included, of whom 78 (40.6%) were tobacco smokers, with a higher smoking prevalence observed in male patients (74/154, 48.1%). The PASI 75 response rates at weeks 4, 8, 12, 24, and 48 were 29.2% (56/192), 54.2% (104/192), 78.6% (151/192), 84.5% (153/181), and 82.7% (134/162), respectively. The PASI 90 response rates increased from 13.0% (25/192) at week 4 to 62.4% (113/181) at week 24 and 59.9% (97/162) at week 48. Logistic regression analysis indicated that nonsmoking patients with psoriasis had a high PASI 75 response rate. The adjusted odds ratios were 2.57 (95% CI 1.19‐5.53), 2.61 (95% CI 1.34‐5.08), 2.62 (95% CI 1.13‐6.04), 2.27 (95% CI 0.89‐5.75), and 2.75 (95% CI 1.01‐7.49) at weeks 4, 8, 12, 24, and 48, respectively. Moreover, nonsmoking patients with psoriasis also had a higher PASI 90 response rate than those who smoked. The odds ratios ranged from 1.32 (95% CI 0.49‐3.54) to 2.59 (95% CI 1.21‐5.55). Correlation analysis showed that both tobacco smoking duration and daily cigarette consumption were negatively correlated with the reduction in PASI score at weeks 4 to 48 after treatment (*P*<.05).

**Conclusions:**

Tobacco smoking was negatively associated with treatment response among patients with psoriasis undergoing biologic treatment, especially among patients with longer tobacco smoking duration and higher daily cigarette consumption.

## Introduction

Psoriasis is a chronic and inflammatory skin disease, and most patients with psoriasis have some detriment to their quality of life [1]. Most patients with mild to moderate psoriasis can control their condition with topical medications or phototherapy, but these may not be sufficient for moderate to severe cases [2]. The emergence of biologics has brought about a major breakthrough in the treatment of psoriasis, particularly playing a positive role in patients with moderate to severe psoriasis. Biologics precisely target certain cytokines in inflammatory pathways during the pathological process of psoriasis, such as tumor necrosis factor, interleukin (IL)-17, and IL-23, to prevent inflammatory reactions and achieve efficient clearance of psoriasis lesions [3,4].

In recent years, guidelines on the treatment of psoriasis in Europe and the United States have shown growing emphasis and stronger recommendations for biologics [2,5]. The guidelines for the diagnosis and treatment of psoriasis in China also increasingly recommend biologics in clinical treatment, and the “Guidelines for the treatment of psoriasis with biologics and small-molecule drugs in China” were published in 2024, providing recommendations for clinicians on the use of biologic therapies [6,7]. With the increasing accessibility of biologics and support from medical insurance policies, more and more patients with psoriasis benefit from biologics. Previous studies have shown that biologics are efficacious in the treatment of psoriasis and are associated with greater improvements in quality of life compared with other treatment modalities [3,8,9]. However, some patients still fail to achieve satisfactory therapeutic responses in clinical practice, and factors such as genetics, obesity, tobacco smoking, alcohol consumption, and disease-related features (eg, disease duration and age at diagnosis) may contribute to the heterogeneity of responses to biologics [10,11].

Tobacco smoking is a widespread unhealthy lifestyle habit, with approximately 1.14 billion current smokers worldwide (approximately 30% in China), resulting in enormous health and economic consequences [12]. The impact of tobacco smoking on health has been extensively studied, and smoking is recognized as being closely associated with the development of various cardiovascular diseases, pulmonary diseases, and cancers [13-15]. The association between tobacco smoking and psoriasis is also receiving increasing attention. Previous studies have demonstrated that tobacco smoking elevates the risk of psoriasis and exacerbates disease severity, making the condition more difficult to control [16-18]. However, the impact of smoking on the efficacy of biologic treatment in patients with psoriasis remains inconclusive. Some studies have reported that smoking has a negative impact on the efficacy of biologic treatment for psoriasis [19-25], whereas other studies have failed to detect a significant effect of smoking on treatment outcomes [26-28]. Therefore, this study aimed to explore the impact of tobacco smoking on the efficacy of biologic treatment among patients with psoriasis in Shanghai, with the expectation of providing valuable insights for clinical practice regarding psoriasis treatment.

## Methods

### Study Population

A hospital-based prospective observational study was conducted in Shanghai Skin Disease Hospital from 2022 to 2024. Patients with psoriasis vulgaris aged ≥18 years, both male and female, and without a migration plan within a year were included and followed up on for 48 weeks, and patients who were unable to provide informed consent or who had neurological or psychiatric disorders were excluded. In this study, we extracted data from 192 patients with psoriasis who received biologic treatment with secukinumab.

### Data Collection

Data were collected through a self-designed questionnaire administered by dermatologists after receiving uniform training. The main contents of the questionnaire were as follows: (1) demographic characteristics, including age, gender, educational level, monthly income, residence, marital status, and BMI; (2) information on tobacco smoking status, including smoking initiation age, smoking duration, and number of daily consumed cigarettes; and (3) information on psoriasis duration and severity. The severity of psoriasis was assessed by dermatologists using the Psoriasis Area and Severity Index (PASI), body surface area (BSA), and Physician Global Assessment (PGA) at baseline, week 4, week 8, week 12, week 24, and week 48.

### Definition and Classification

In this study, the age of patients with psoriasis was classified as <35, 35 to 45, 46 to 60, and >60 years. Educational level was categorized into 4 groups (primary school or lower, junior high school, senior high school, and college or higher) based on the highest level of education completed. Monthly income (in CNY) was categorized into <¥3000, ¥3000 to ¥5000, ¥5001 to ¥10,000, and >¥10,000 (¥1=US $0.14). Residence was classified as urban and rural, and marital status was classified as married; unmarried; and divorced, widow or widower, or other. The BMI of patients with psoriasis was categorized as <23.9 kg/m^2^ (lower or normal weight), 24.0 to 28.0 kg/m^2^ (overweight), and >28.0 kg/m^2^ (obesity).

A smoker was defined as a person who had smoked at least 100 cigarettes in their lifetime. Smoking initiation age was classified as <25 years and ≥25 years. Smoking duration was calculated as the age at the time of the study minus the smoking initiation age and then stratified into <20 years and ≥20 years. The number of daily consumed cigarettes was categorized as <20 and ≥20.

The PASI 75, PASI 90, and PGA (1/0) rate were calculated and used to evaluate the treatment response. The PASI 75 and PASI 90 were defined as patients with a reduction in PASI score of ≥75% and ≥90%, respectively, calculated using the formula [(PASI at baseline – PASI at week *t*)/PASI at baseline] × 100%. The PGA (1/0) rate referred to the proportion of patients with a PGA score of 1 or 0 among all patients.

### Statistical Analysis

Data analysis was performed using the SAS software (version 9.4; SAS Institute). Descriptive statistics were presented as means and SDs or medians and IQRs as appropriate for quantitative variables and frequency counts and proportions for qualitative variables. The 2-tailed Student *t* test, Mann-Whitney *U* test, or Pearson chi-square test was performed to examine the statistical difference between groups. The temporal trend of treatment response (PASI 75, PASI 90, and PGA [1/0]) at different weeks among patients with psoriasis was evaluated using the Cochran‐Armitage trend test. Logistic regression was applied to calculate the odds ratios (ORs) and 95% CIs to explore the association between tobacco smoking and treatment response among patients with psoriasis at weeks 4, 8, 12, 24, and 48. Scatterplots and quadratic polynomial regression were used to explore the association among smoking duration, daily cigarette consumption, and PASI score reduction. A *P* value of less than .05 (2 tailed) was considered statistically significant.

### Ethical Considerations

This study was approved by the institutional review board of Shanghai Skin Disease Hospital (2022-25). Informed consent was obtained before starting the study, and the study was strictly performed in accordance with the Declaration of Helsinki. Patient participation in this study was voluntary, and patients could withdraw at any time. Participants’ privacy and confidentiality were strictly protected. All data were deidentified before analysis and were accessible only to the researchers of this study. No compensation was provided to participants.

## Results

### Overview

A total of 192 patients with psoriasis undergoing biologic treatment were included in the final data analysis, of whom 154 (80.2%) were male. The average age of the patients was 41.2 (SD 18.2) years, with 41.1% (n=79) of patients aged >45 years. Among the patients, 49.0% (n=94) had a college or higher educational level, whereas 56.8% (n=109) reported monthly incomes exceeding ¥5000. Approximately 66.7% (n=128) of patients were urban residents, and 58.9% (n=113) of them were married. The average BMI of patients was 25.8 (SD 4.3) kg/m^2^, and 65.1% (n=125) were overweight or obese. The median disease duration was 13.5 (IQR 7.0-21.0) years, and the median values of the PASI, BSA, and PGA scores at baseline (week 0) were 12.2 (IQR 9.8-18.6), 18.5 (IQR 12.0-33.3), and 3.0 (IQR 2.0-3.0), respectively. Data in Table 1 indicate that female patients were older (*t*_190_=–2.64; *P*=.009) and had higher educational levels (*χ*^2^_1_=5.4; *P*=.02), higher incomes (*χ*^2^_3_=15.2; *P*=.002), and longer disease duration (*z*=–2.18; *P*=.03), and there was a higher proportion of married individuals in this group (*χ*^2^_2_=11.8; *P*=.003) but a lower proportion of overweight or obesity (*χ*^2^_2_=11.1; *P*=.004) and lower PGA scores than among male patients (*z*=–3.73; *P*<.001). The differences were all statistically significant (*P*<.05; Table 1).

**Table 1.** The baseline characteristics of patients with psoriasis undergoing biologic treatment (N=192).

Characteristic	Total	Male patients (n=154)	Female patients (n=38)	*P* value
Age (y), mean (SD)	41.2 (18.2)	39.5 (18.5)	48.1 (15.4)	.009
Age group (y), n (%)				.04
<35	76 (39.6)	67 (43.5)	9 (23.7)	
35-45	37 (19.3)	28 (18.2)	9 (23.7)	
46-60	42 (21.9)	32 (20.8)	10 (26.3)	
>60	37 (19.3)	27 (17.5)	10 (26.3)	
Educational level, n (%)				.02
Primary school or lower	48 (25.0)	45 (29.2)	3 (7.9)	
Junior high school	25 (13.0)	19 (12.3)	6 (15.8)	
Senior high school	25 (13.0)	19 (12.3)	6 (15.8)	
College or higher	94 (49.0)	71 (46.1)	23 (60.5)	
Monthly income (¥; ¥1=US $0.14), n (%)				.002
<3000	47 (24.5)	45 (29.2)	2 (5.3)	
3000-5000	36 (18.8)	32 (20.8)	4 (10.5)	
5001-10,000	101 (52.6)	71 (46.1)	30 (78.9)	
>10,000	8 (4.2)	6 (3.9)	2 (5.3)	
Residence, n (%)				.16
Urban	128 (66.7)	99 (64.3)	29 (76.3)	
Rural	64 (33.3)	55 (35.7)	9 (23.7)	
Marital status, n (%)				.003
Married	113 (58.9)	82 (53.2)	31 (81.6)	
Unmarried	48 (25.0)	46 (29.9)	2 (5.3)	
Divorced, widow or widower, or other	31 (16.1)	26 (16.9)	5 (13.2)	
BMI (kg/m^2^), mean (SD)	25.8 (4.3)	26.4 (4.1)	23.5 (4.4)	<.001
BMI (kg/m^2^), n (%)				.004
<23.9 (low or normal weight)	67 (34.9)	45 (29.2)	22 (57.9)	
24.0-28.0 (overweight)	73 (38.0)	63 (40.9)	10 (26.3)	
>28.0 (obesity)	52 (27.1)	46 (29.9)	6 (15.8)	
Disease duration (y), median (IQR)	13.5 (7.0-21.0)	12.5 (7.0-20.0)	19.5 (11.0-25.0)	.03
PASI^a^ at week 0, median (IQR)	12.2 (9.8-18.6)	12.3 (10.0-19.2)	11.3 (8.0-16.8)	.30
BSA^b^ at week 0 (%), median (IQR)	18.5 (12.0-33.3)	18.5 (12.0-32.0)	16.8 (11.0-33.5)	.62
PGA^c^ at week 0, median (IQR)	3.0 (2.0-3.0)	3.0 (2.0-3.0)	2.2 (2.0-2.7)	<.001

a PASI: Psoriasis Area and Severity Index. The PASI indicates the severity of psoriasis, with a higher value representing a more severe condition (0-72).

b BSA: body surface area. BSA indicates the affected body surface, with a higher value representing a more severe condition (0%-100%).

c PGA: Physician Global Assessment. The PGA indicates the physician’s overall evaluation of psoriasis, with a higher score representing a more severe condition (0-5).

### Tobacco Smoking Among Patients With Psoriasis

As shown in Table 2, of the 192 patients with psoriasis undergoing biologic treatment, 78 (40.6%) were tobacco smokers, including 74 (94.9%) male and 4 (5.1%) female patients. The prevalence of tobacco smoking was higher in male patients (74/154, 48.1%) than in female patients (4/38, 10.5%), and the difference was statistically significant (*χ*^2^_1_=17.8; *P*<.001).

**Table 2.** Tobacco smoking characteristics among patients with psoriasis (N=192).

Variable	Total	Male patients (n=154)	Female patients (n=38)	*P* value
Tobacco smoking, n/N (%)				<.001
Yes	78/192 (40.6)	74/154 (48.1)	4/38 (10.5)	
No	114/192 (59.4)	80/154 (51.9)	34/38 (89.5)	
Smoking initiation age (y), mean (SD)	24.0 (4.9)	24.0 (4.8)	24.0 (6.7)	.99
Smoking initiation age (y), median (IQR)	24.0 (20.0-26.0)	24.5 (20.0-26.0)	21.0 (20.0-28.0)	.67
Smoking initiation age (y), n/N (%)				.64
<25	40/78 (51.3)	37/74 (50.0)	3/4 (75.0)	
≥25	38/78 (48.7)	37/74 (50.0)	1/4 (25.0)	
Smoking duration (y), mean (SD)	21.2 (16.1)	20.5 (15.7)	32.8 (21.6)	.14
Smoking duration (y), median (IQR)	20.0 (9.0-35.0)	19.0 (9.0-35.0)	35.0 (14.5-51.0)	.21
Smoking duration (y), n/N (%)				.64
<20	38/78 (48.7)	37/74 (50.0)	1/4 (25.0)	
≥20	40/78 (51.3)	37/74 (50.0)	3/4 (75.0)	
Cigarettes consumed per d, mean (SD)	16.9 (7.9)	17.1 (8.1)	13.8 (4.8)	.42
Cigarettes consumed per d, median (IQR)	20.0 (10.0-20.0)	20.0 (10.0-20.0)	12.5 (10.0-17.5)	.30
Cigarettes consumed per d, n/N (%)				.34
<20	31/78 (39.7)	28/74 (37.8)	3/4 (75.0)	
≥20	47/78 (60.3)	46/74 (62.2)	1/4 (25.0)	

The average and median age of smoking initiation was 24.0 (SD 4.9; IQR 20.0-26.0) years, and 51.3% (40/78) of patients began tobacco use at <25 years. The mean smoking duration was 21.2 (SD 16.1) years, and the median duration was 20.0 (IQR 9.0-35.0) years. A total of 51.3% (40/78) of patients had smoked for more than 20 years. The mean daily cigarette consumption among patients was 16.9 (SD 7.9) cigarettes, with a median of 20 (IQR 10.0-20.0) cigarettes. In total, 60.3% (47/78) of patients smoked more than 20 cigarettes per day. The differences between male and female patients who smoked tobacco in smoking initiation age (*t*_76_=0.01 and *P*=.99; *z*=–0.42 and *P*=.67; *χ*^2^_1_=0.2 and *P*=.64), smoking duration (*t*_76_=–1.48 and *P*=.14; *z*=–1.26 and *P*=.21; *χ*^2^_1_=0.2 and *P*=.64), and daily cigarette consumption (*t*_76_=0.80 and *P*=.42; *z*=–1.04 and *P*=.30; *χ*^2^_1_=0.9 and *P*=.34) were not statistically significant (Table 2).

### Treatment Outcomes Among Patients With Psoriasis

Table 3 illustrates the longitudinal outcomes of PASI, BSA, and PGA scores, along with the achievement of PASI 75, PASI 90, and PGA (1/0) at weeks 4, 8, 12, 24, and 48 after treatment. During the treatment period, the achievement of PASI 75, PASI 90, and PGA (1/0) showed an increasing trend (*Z*=9.42 and *P*<.001; *Z*=8.53 and *P*<.001; and *Z*=3.14 and *P*=.002, respectively). The response rate increased from 29.2% (56/192) at week 4 to 82.7% (134/162) at week 48 for PASI 75, from 13.0% (25/192) at week 4 to 59.9% (97/162) at week 48 for PASI 90, and from 47.4% (91/192) at week 4 to 69.8% (113/162) at week 48 for PGA (1/0; Table 3).

**Table 3.** Treatment outcomes among patients with psoriasis at weeks 4, 8, 12, 24, and 48.

Variable	Weeks after treatment				
	Week 4 (n=192)	Week 8 (n=192)	Week 12 (n=192)	Week 24 (n=181)	Week 48 (n=162)
PASI^a^					
Mean (SD)	6.7 (6.3)	4.3 (5.1)	2.2 (3.1)	1.6 (2.9)	1.9 (3.0)
Median (IQR)	4.7 (2.1-10.2)	2.5 (0.8-5.7)	1.2 (0.0-3.0)	0.4 (0.0-1.8)	0.6 (0.0-2.4)
PASI 75, n (%)	56 (29.2)	104 (54.2)	151 (78.6)	153 (84.5)	134 (82.7)
PASI 90, n (%)	25 (13.0)	57 (29.7)	104 (54.2)	113 (62.4)	97 (59.9)
BSA^b^ (%)					
Mean (SD)	14.9 (15.9)	8.9 (12.1)	3.8 (7.9)	1.6 (2.8)	2.5 (6.0)
Median (IQR)	10.0 (4.0-19.8)	5.0 (1.0-12.5)	1.1 (0.0-4.0)	0.4 (0.0-1.8)	0.2 (0.0-2.5)
PGA^c^					
Mean (SD)	1.6 (0.9)	1.2 (1.0)	0.9 (1.0)	0.9 (1.0)	1.1 (1.1)
Median (IQR)	2.0 (1.0-2.0)	1.0 (0.0-2.0)	1.1 (0.0-1.0)	1.0 (0.0-1.0)	1.0 (0.0-2.0)
PGA (1/0), n (%)	91 (47.4)	124 (64.6)	145 (75.5)	136 (75.1)	113 (69.8)

a PASI: Psoriasis Area and Severity Index. Indicates the severity of psoriasis, with a higher value representing a more severe condition (0-72).

b BSA: body surface area. Indicates the affected body surface, with a higher value representing a more severe condition (0%-100%).

c PGA: Physician Global Assessment. Indicates the physician’s overall evaluation of psoriasis, with a higher score representing a more severe condition (0-5).

**Figure 1.**
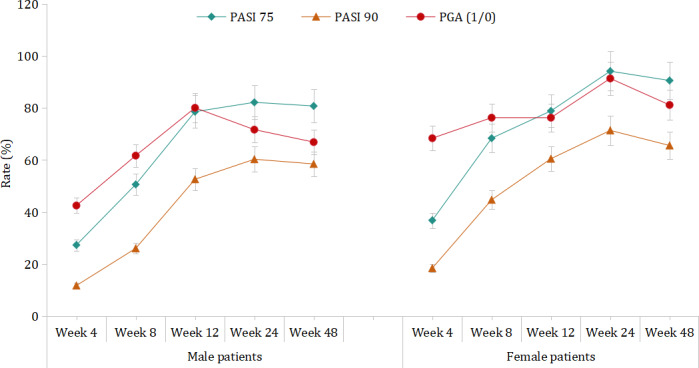
Treatment responses (Psoriasis Area and Severity Index [PASI] 75, PASI 90, and Physician Global Assessment [PGA] [1/0]) of male and female patients at weeks 4, 8, 12, 24, and 48.

Stratification by gender revealed that, in male patients, the PASI 75, PASI 90, and PGA (1/0) response rates all showed an increasing trend (*Z*=8.35 and *P*<.001; *Z*=7.84 and *P*<.001; and *Z*=2.85 and *P*=.004, respectively). In female patients, only the PASI 75 and PASI 90 response rates increased with biologic treatment at different weeks (*Z*=4.47 with *P*<.001 and *Z*=3.47 with *P*<.001, respectively) but not the PGA (1/0) response rate (*Z*=1.42; *P*=.16; Figure 1). Moreover, data in Figure 1 also indicate that female patients typically achieved better response rates (PASI 75, PASI 90, and PGA [1/0]) than male patients at the same evaluation time points.

### Association Between Tobacco Smoking and Treatment Response

Logistic regression analysis indicated that patients with psoriasis who did not smoke tobacco had higher PASI 75 response rates than those who smoked tobacco, and the ORs were 2.57 (95% CI 1.19-5.53), 2.61 (95% CI 1.34-5.08), 2.62 (95% CI 1.13-6.04), 2.27 (95% CI 0.89-5.75), and 2.75 (95% CI 1.01-7.49) at weeks 4, 8, 12, 24, and 48, respectively, even with the adjustment of potential confounders. Patients who did not smoke tobacco also had higher PASI 90 response rates than those who smoked. The ORs were 1.32 (95% CI 0.49-3.54), 1.78 (95% CI 0.85-3.71), 1.71 (95% CI 0.88-3.33), 1.38 (95% CI 0.70-2.71), and 2.59 (95% CI 1.22-5.55) at weeks 4, 8, 12, 24, and 48, respectively. For PGA (1/0), patients who did not smoke tobacco tended to have higher response rates than those who smoked tobacco at weeks 4, 8, 12, 24, and 48. The ORs were 1.08 (95% CI 0.57-2.05), 1.14 (95% CI 0.58-2.24), 1.55 (95% CI 0.70-3.39), 1.52 (95% CI 0.69-3.31), and 1.18 (95% CI 0.54-2.59), respectively (Table 4).

**Table 4.** The association between tobacco smoking and treatment response (Psoriasis Area and Severity Index [PASI] 75, PASI 90, and Physician’s Global Assessment [PGA] [1/0]) among patients with psoriasis at weeks 4 to 48.

Variable	Response rate (nonsmokers vs smokers; %)	OR^a^ (95% CI)		
		Model A^b^	Model B^c^	Model C^d^
PASI 75				
Week 4	36.8 vs 17.9	2.67 (1.34-5.33)	2.56 (1.23-5.31)	2.57 (1.19-5.53)
Week 8	64.9 vs 38.5	2.96 (1.63-5.38)	2.54 (1.35-4.78)	2.61 (1.34-5.08)
Week 12	84.2 vs 70.5	2.23 (1.11-4.49)	2.23 (1.04-4.79)	2.62 (1.13-6.04)
Week 24	90.4 vs 76.6	2.87 (1.24-6.64)	2.33 (0.96-5.67)	2.27 (0.89-5.75)
Week 48	89.8 vs 74.3	3.03 (1.28-7.20)	2.72 (1.07-6.91)	2.75 (1.01-7.49)
PASI 90				
Week 4	14.9 vs 10.3	1.53 (0.63-3.75)	1.33 (0.50-3.51)	1.32 (0.49-3.54)
Week 8	36.0 vs 20.5	2.18 (1.11-4.25)	1.80 (0.88-3.69)	1.78 (0.85-3.71)
Week 12	59.6 vs 46.2	1.72 (1.00-3.09)	1.67 (0.90-3.11)	1.71 (0.88-3.33)
Week 24	66.3 vs 57.1	1.48 (0.81-2.72)	1.31 (0.68-2.51)	1.38 (0.70-2.71)
Week 48	68.2 vs 50.0	2.14 (1.13-4.06)	2.30 (1.14-4.66)	2.59 (1.21-5.55)
PGA (1/0)				
Week 4	51.3 vs 42.3	1.44 (0.80-2.57)	1.10 (0.59-2.07)	1.08 (0.57-2.05)
Week 8	67.5 vs 60.3	1.37 (0.75-2.50)	1.13 (0.60-2.14)	1.14 (0.58-2.24)
Week 12	78.2 vs 74.3	1.23 (0.63-2.45)	1.43 (0.69-2.99)	1.55 (0.70-3.39)
Week 24	80.8 vs 68.4	1.94 (0.98-3.85)	1.55 (0.74-3.22)	1.52 (0.69-3.31)
Week 48	72.7 vs 66.2	1.36 (0.70-2.67)	1.21 (0.58-2.50)	1.18 (0.54-2.59)

a OR: odds ratio.

b Model A: univariate logistic regression.

c Model B: multivariate logistic regression with adjustment for age and sex.

d Model C: multivariate logistic regression with adjustment for age, educational level, sex, marital status, monthly income, and BMI.

**Figure 2.**
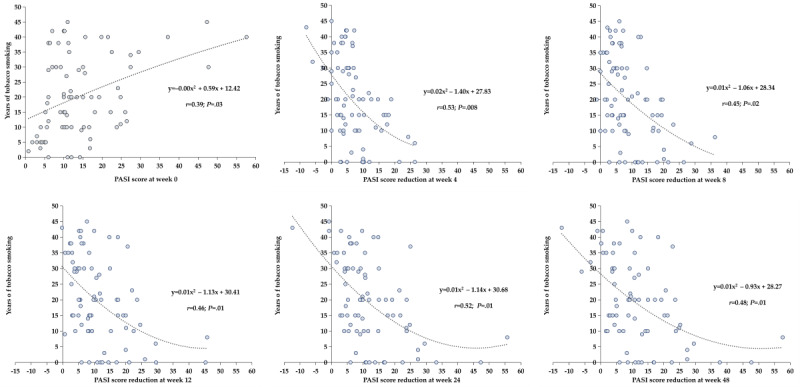
Correlation analysis between the Psoriasis Area and Severity Index (PASI) score at week 0 or PASI score reduction at weeks 4 to 48 and the years of tobacco smoking among smokers.

**Figure 3.**
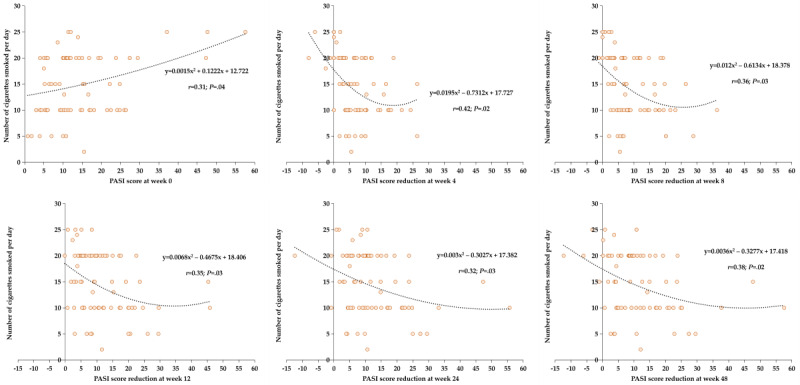
Correlation analysis between the Psoriasis Area and Severity Index (PASI) score at week 0 or PASI score reduction at weeks 4 to 48 and the number of cigarettes smoked per day.

Among the 40.6% (78/192) of patients with psoriasis who smoked tobacco, the PASI score at baseline was positively correlated with tobacco smoking years (*r*=0.39; *P*=.03) and daily cigarette consumption (*r*=0.31; *P*=.04). Moreover, the PASI score reduction at weeks 4, 8, 12, 24, and 48 from baseline (week 0) was negatively correlated with tobacco smoking years (Figure 2) and daily cigarette consumption (Figure 3).

## Discussion

### Principal Findings

This study explored the impact of tobacco smoking on the treatment efficacy of biologics in 192 patients with psoriasis based on real-world clinical data. In this study, the smoking prevalence among patients with psoriasis was 40.6% (78/192), which was consistent with that in previous studies [29-31]. The findings of this study indicated that patients with psoriasis who did not smoke had better treatment response than those who smoked, especially in the PASI 75 evaluation. Moreover, the reduction in PASI score at weeks 4, 8, 12, 24, and 48 from baseline was negatively correlated with both tobacco smoking years and number of daily cigarettes consumed.

Rapid clearance of skin lesions is the greatest treatment need among patients with psoriasis in China [32]. The emergence and application of biologics have brought about a qualitative leap in the treatment goals for psoriasis, with PASI 90 and PASI 100 becoming the new therapeutic targets for the biologic treatment of psoriasis [2,5,6]. In a previous study, based on data from the Psoriasis Study of Health Outcomes, Lynde et al [33] reported that the response rates for the primary end point (static PGA [0/1] and/or PASI 90) at week 12 were 71.4% and 58.6% for patients receiving anti–IL-17A biologics and other biologics, respectively. Pinter et al [34] compared the long-term treatment effectiveness of different biologics, including ixekizumab, risankizumab, and ustekinumab, and indicated that the response rates for PASI 90 were 40.5% to 64.3% at month 6 and 41.7% to 61.4% at month 12. In this study, we observed similar results, with overall PASI 75 response rates of 78.6% (151/192), 84.5% (153/181), and 82.7% (134/162); PASI 90 response rates of 54.2% (104/192), 62.4% (113/181), and 59.9% (97/162); and PGA (1/0) rates of 75.5% (145/192), 75.1% (136/181), and 69.8% (113/162) at weeks 12, 24, and 48, respectively. The response rates for PASI 75 and PASI 90 at week 24 were slightly higher than those at week 48, which might be attributed to an increased rate of treatment discontinuation after receiving 24 weeks of treatment among patients.

Tobacco smoking is recognized as an important risk factor for psoriasis [35,36]. In this study, we noticed that tobacco smoking was negatively associated with treatment response at weeks 4, 8, 12, 24, and 48 among patients with psoriasis undergoing treatment with biologics. Compared to patients who smoked tobacco, patients who did not smoke tobacco had 2.75 and 2.59 times higher odds of achieving PASI 75 and PASI 90 at week 48, respectively, which is consistent with most previous studies. Two meta-analyses have reported that patients with psoriasis who smoke exhibit poorer response to biologics [20,23]. In a multicenter longitudinal cohort study, Warren et al [24] found that smokers had reduced odds of achieving PASI 90 at 6 and 12 months after initiating biologic therapies. These studies all suggest a negative impact of tobacco smoking on treatment response to biologics for psoriasis.

Previous studies have indicated that smoking intensity and duration are positively correlated with the severity of psoriasis in a dose-dependent fashion [17,18,37]. In this study, we observed consistent results demonstrating that PASI score was positively correlated with tobacco smoking years and number of daily consumed cigarettes. Moreover, we focused on the relationship between smoking and treatment efficacy among patients with psoriasis undergoing treatment with biologics during the 48-week follow-up period. The results showed that the reduction in PASI score was negatively correlated with tobacco smoking duration and tobacco smoking intensity. However, the specific mechanism of how tobacco smoking affects the efficacy of biologics for psoriasis still requires further research and exploration.

In this study, we also noticed that female patients had better treatment responses than male patients in the PASI 75, PASI 90, and PGA (1/0). There are several possible explanations for this gender disparity. First, female patients in this study had lower disease severity than male patients at baseline, which may have made it easier for female patients to achieve disease improvement. Second, BMI may also play a role in this difference. In this study, the average BMI of female patients was significantly lower than that of male patients, and higher BMI is considered to be associated with increased disease severity and reduced treatment response in psoriasis [38]. Pharmacokinetic studies have shown that excessive body weight can have a negative impact on drug clearance and volume of distribution [38]. Third, the proportion of female patients with a college education or higher was higher than that of male patients, and a higher educational level often leads to a better treatment adherence and a subsequently better treatment response [39]. Fourth, the lower tobacco smoking proportion might have also contributed to the better treatment response among female patients with psoriasis.

A key strength of this study was that the clinical data of patients with psoriasis were extracted directly from the health information system, eliminating recall bias, which ensured high data quality. The collection of data for over 48 weeks, allowing for the assessment of the impact of tobacco smoking on biologic efficacy at multiple time points, was another strength. Additionally, we used a variety of evaluation tools to assess patient outcomes, including the PASI, BSA, and PGA, thereby providing more and multidimensional data support for the study results.

### Limitations

This study has several limitations. First, the 192 patients were recruited from only 1 hospital, which ensured high internal authenticity; however, the generalization of the findings to other patients with psoriasis is limited. Second, the smoking status of patients was self-reported, which may be subject to social desirability bias and recall bias. The lack of information on secondhand smoke and e-cigarette exposure may have underestimated the impact of smoking on treatment efficacy to some extent. Third, the number of female patients included in this study was relatively low, especially among smokers, which may have resulted in the findings more strongly reflecting the situation of male patients. Future studies should consider conducting investigations with larger sample sizes to comprehensively assess the impact of smoking on treatment response in patients with psoriasis with different demographic characteristics.

### Conclusions

The prevalence of tobacco smoking among patients with psoriasis undergoing biologic treatment was high, especially among male patients. Tobacco smoking was negatively associated with treatment response among patients with psoriasis undergoing biologic treatment, especially among those with longer tobacco smoking duration and higher daily cigarette consumption. Therefore, we recommend that dermatologists actively assess tobacco smoking status among patients and encourage them to quit smoking to improve their treatment response.

## Abbreviations

BSA: body surface area
IL: interleukin
OR: odds ratio
PASI: Psoriasis Area and Severity Index
PGA: Physician Global Assessment
